# Artificial Intelligence in Healthcare: From Diagnosis to Rehabilitation

**DOI:** 10.7759/cureus.102286

**Published:** 2026-01-25

**Authors:** Karolina Witek, Marta Nowocien, Joanna Gerlach, Natalia Guzik, Barbara Balajewicz, Lukasz Siwek, Karolina Lichwala, Oliwia Sipiora, Jakub Andrzejewicz, Monika Chlipala

**Affiliations:** 1 General Medicine, Specialist Hospital Named After H. Klimontowicz in Gorlice, Gorlice, POL; 2 General Medicine, Municipal Hospital in Zabrze, Zabrze, POL; 3 General Medicine, University Teaching Hospital Them F. Chopin in Rzeszów, Rzeszów, POL; 4 General Medicine, Medical University of Silesia, Katowice, POL; 5 General Medicine, Medical University of Silesia, Zabrze, POL; 6 Paediatrics, St. Luke’s Voivodeship Hospital in Tarnów, Tarnów, POL; 7 General Medicine, Health Care Complex in Dąbrowa Tarnowska (ZOZ DT), Dąbrowa Tarnowska, POL; 8 General Medicine, St. Luke's Voivodeship Hospital in Tarnów, Tarnów, POL

**Keywords:** artificial intelligence, chatbots, clinical decision support systems, diagnostic imaging, digital health, laboratory medicine, machine learning, medical diagnostics, rehabilitation

## Abstract

Artificial intelligence (AI) is increasingly integrated into modern healthcare, with rapidly expanding applications in medical diagnostics, laboratory medicine, rehabilitation, and patient-centered digital health solutions. The aim of this narrative review is to provide a critically curated overview of current clinical applications of AI across the healthcare continuum, from diagnosis to rehabilitation, while highlighting their clinical benefits, limitations, and implementation challenges.

A targeted narrative literature search was conducted using major biomedical databases, including PubMed/MEDLINE, Scopus, Web of Science, and Embase, with emphasis on recent and influential studies published primarily over the past decade. Evidence was qualitatively synthesized across key clinical domains, including diagnostic imaging, laboratory diagnostics, rehabilitation technologies, and conversational agents.

The reviewed literature indicates that AI systems can achieve diagnostic performance comparable to healthcare professionals in selected, well-defined tasks, particularly within imaging-based specialties such as radiology, mammography, ophthalmology, dermatology, and digital pathology, predominantly under retrospective or controlled study conditions. In laboratory medicine, AI-based tools support workflow optimization, result interpretation, and clinical decision support, while in rehabilitation, AI-enabled systems - including robotics, motion analysis platforms, and large language models - facilitate personalized therapy and functional recovery, albeit with heterogeneous evidence and limited prospective validation. AI-based chatbots demonstrate potential to support patient education, mental health interventions, and communication workflows, particularly as adjuncts to clinician-led care.

Despite these advances, challenges related to generalizability, algorithmic bias, ethical implementation, and regulatory oversight persist. Overall, this review underscores that AI should be regarded as a supportive clinical decision-support technology rather than a replacement for healthcare professionals, with future research prioritizing prospective validation, real-world effectiveness, and responsible integration into routine clinical practice.

## Introduction and background

Artificial intelligence (AI) has emerged as one of the most influential technological developments in contemporary medicine and is increasingly regarded as a foundational component of future healthcare systems [1]. The ability of AI-based systems to analyze large volumes of complex data, recognize patterns, and support clinical decision-making has positioned these technologies as promising tools across a wide range of medical disciplines [2,3]. In particular, medical diagnostics, which rely heavily on image interpretation, signal analysis, and multimodal data integration, have become a primary area for AI implementation [4-6].

Global healthcare systems face mounting challenges, including population aging, an increasing burden of chronic diseases, and persistent shortages of qualified healthcare professionals [1,7]. These pressures have intensified the demand for solutions that improve efficiency, enhance diagnostic accuracy, and optimize the allocation of limited resources. AI-based technologies have therefore been proposed as potential enablers of system-level improvements by supporting clinicians, reducing diagnostic errors, and streamlining clinical workflows, particularly in high-volume and resource-constrained clinical settings [1,2,7].

Evidence from systematic reviews and meta-analyses suggests that, in selected and well-defined diagnostic tasks, particularly within imaging-based specialties, AI algorithms can achieve performance comparable to that of healthcare professionals, predominantly under retrospective or controlled study conditions [4,8]. However, most available studies focus on technical performance metrics rather than real-world clinical outcomes, and frequently lack external validation across diverse populations and healthcare environments, limiting the generalizability of these findings [4,8]. Consequently, the translation of AI systems into routine clinical practice remains complex and requires careful validation, clinician oversight, and contextualization within existing care pathways.

Beyond diagnostics, increasing attention has been directed toward the application of AI in laboratory medicine, rehabilitation, and patient-facing digital health tools, including conversational agents [9-15]. These applications reflect a broader shift toward data-driven, personalized, and technology-assisted healthcare models, with potential implications for workflow efficiency, patient engagement, and continuity of care. The evidence base supporting these applications - derived from systematic reviews, randomized controlled trials, and key observational studies across diagnostic imaging, laboratory medicine, rehabilitation, and digital health - is summarized in tables in the subsequent sections [1-28]. At the same time, the rapid expansion of AI in medicine has raised important ethical, legal, and regulatory concerns related to algorithm transparency, explainability, accountability, data privacy, and patient safety [16,17]. Regulatory frameworks governing medical AI are still evolving and must address the unique challenges posed by adaptive, learning algorithms deployed in high-risk clinical settings [17].

The aim of this narrative review is to provide a critically curated overview of clinically relevant applications of AI across the healthcare continuum, from medical diagnostics to rehabilitation and patient support. The review focuses on domains in which AI has progressed beyond proof-of-concept toward clinical evaluation, emphasizing clinical utility, implementation considerations, and limitations of the current evidence base rather than technical development alone. A narrative synthesis approach based on a targeted literature search of major biomedical databases was adopted to contextualize current evidence and identify key challenges and future research directions.

## Review

Materials and methods

This article was conducted as a narrative review aimed at providing a critically curated overview of clinically relevant applications of AI in healthcare. A targeted literature search was performed to identify recent and influential publications addressing the use of AI in medical diagnostics, laboratory medicine, rehabilitation, and patient-centered digital health.

The literature search was conducted using the following electronic databases: PubMed/MEDLINE, Scopus, Web of Science, and Embase. The search covered studies published between January 2015 and December 2025 and was limited to articles published in English. Search strategies combined controlled vocabulary and free-text terms related to AI and healthcare applications, including, but not limited to: “artificial intelligence,” “machine learning,” “deep learning,” “medical diagnostics,” “radiology,” “laboratory medicine,” “rehabilitation,” “clinical decision support,” and “digital health.” Database-specific adaptations of these terms were applied as appropriate.

Inclusion criteria comprised peer-reviewed articles focusing on clinical or clinically relevant applications of AI in human healthcare, including systematic reviews, meta-analyses, randomized controlled trials, observational studies, and selected high-quality narrative reviews. Studies were excluded if they were limited to purely technical algorithm development without clinical context, animal studies, non-healthcare applications, conference abstracts without full text, or opinion pieces lacking substantive reference to empirical evidence.

Study selection was performed by the authors through an iterative screening of titles and abstracts, followed by full-text assessment of potentially relevant articles. Publications were prioritized based on clinical relevance, methodological rigor, and influence within the field, with particular emphasis on systematic reviews, meta-analyses, and landmark clinical studies where available. Given the narrative nature of the review and the heterogeneity of study designs, no formal quantitative synthesis was conducted.

Qualitative synthesis was performed through thematic grouping of studies according to clinical domain (e.g., diagnostic imaging, laboratory diagnostics, rehabilitation, and digital health), AI application type, and reported clinical implications. Evidence was summarized descriptively, with attention to study design, clinical setting, and reported limitations. A formal risk-of-bias or quality assessment was not undertaken, which is acknowledged as a methodological limitation of this narrative review, particularly when interpreting claims related to AI performance and clinical effectiveness.

Applications of AI in medical diagnostics

AI represents one of the most dynamically developing areas of contemporary medicine and is increasingly recognized as a key component of future healthcare systems [1,2]. AI has gained particular importance in medical diagnostics, which largely relies on the analysis of complex data, pattern recognition, and the interpretation of medical images and biological signals. Advances in machine learning (ML), especially deep learning (DL), combined with the growing availability of medical data and increased computational power, have enabled the development of algorithms achieving high diagnostic performance [1,4].

Healthcare systems worldwide are facing growing challenges, including population aging, an increasing prevalence of chronic diseases, and shortages of qualified medical personnel. In this context, AI-based diagnostic tools are perceived as potential solutions to improve clinical efficiency, enhance diagnostic quality, and reduce diagnostic errors [2]. At the same time, the implementation of AI in clinical practice raises important questions regarding safety, algorithm generalizability, and its impact on clinical decision-making. A comparative overview of key clinical applications of AI across diagnostic imaging, laboratory medicine, rehabilitation, and patient-centered digital health is summarized in Table 1.

**Table 1:** Clinical applications of artificial intelligence (AI) across the healthcare continuum. Original table created by the authors based on published literature. Note: Evidence across clinical domains is heterogeneous and should be interpreted in the context of study design and clinical setting. RCTs: randomized controlled trials

Clinical domain	Key AI applications	Reported clinical benefits	Evidence type/level	Key limitations/challenges	References
Radiology	Lesion detection, image segmentation, triage	Improved efficiency, reduced reading time	Systematic reviews, retrospective validation studies	Limited external validation, workflow integration	[4,8]
Mammography	Breast cancer screening, double-reading support	Increased cancer detection rate, fewer false positives	Large population-based studies, prospective evaluations	Population heterogeneity, breast density variability	[21]
Ophthalmology	Diabetic retinopathy screening	Early detection, standardized assessment	Screening-based studies, meta-analyses	Image quality dependency, device variability	[22,23]
Dermatology	Skin lesion classification	Improved triage, support in primary care	Benchmark image datasets, retrospective studies	Skin tone bias, image acquisition variability	[24,25]
Digital pathology	Tumor detection, grading, micrometastasis identification	Reduced inter-observer variability	Retrospective slide analysis studies	Workflow integration, regulatory approval	[5,6]
Laboratory diagnostics	Result interpretation, workflow optimization	Faster turnaround time, improved precision	Observational and prospective cohort studies	Data quality, system interoperability	[9-11]
Rehabilitation	Robotic therapy, motion analysis, personalized programs	Enhanced recovery, patient engagement	RCTs, systematic reviews	Heterogeneous outcomes, limited long-term data	[12-14,29]
Chatbots/digital health	Patient education, mental health support	Improved access, scalable interventions	RCTs, observational studies	Limited personalization, adherence concerns	[15]

Technological foundations of AI in diagnostics

Most applications of AI in medical diagnostics are based on supervised ML approaches, in which algorithms are trained using large datasets annotated by clinical experts. Among these methods, convolutional neural networks (CNNs) have become the dominant architecture in diagnostic imaging, as they enable automated extraction of hierarchical image features without the need for manual feature engineering [5]. CNN-based models are widely applied in radiology, mammography, digital pathology, dermatology, and ophthalmology, where they support tasks such as lesion detection, image segmentation, and disease classification.

More recently, transformer-based architectures and multimodal AI models have gained increasing attention. These approaches allow the integration of heterogeneous data sources, including medical images, clinical records, laboratory results, and demographic information, thereby more closely reflecting real-world diagnostic workflows, which rarely rely on a single data modality [6]. For example, multimodal models combining imaging data with clinical variables have demonstrated improved diagnostic performance and risk stratification in selected clinical scenarios.

Despite their technical advances, AI diagnostic systems face important limitations that affect their clinical applicability. Model performance is highly dependent on the quality, diversity, and representativeness of training datasets, and algorithms trained on curated or single-center data may demonstrate reduced accuracy when deployed in new clinical environments. In addition, many high-performing models lack interpretability, functioning as “black-box” systems that provide limited insight into decision-making processes, which may hinder clinical trust and accountability. External validation across diverse populations and careful integration into clinical workflows, therefore, remain essential prerequisites for safe and effective implementation of AI-based diagnostic tools [4].

AI in diagnostic imaging

Radiology

Radiology represents one of the most extensively studied domains of AI implementation in medical diagnostics. DL algorithms, particularly CNNs, are widely applied to tasks such as the detection of neoplastic lesions, intracranial hemorrhage, pneumonia, pulmonary embolism, and interstitial lung disease across modalities, including computed tomography, magnetic resonance imaging, and radiography [7,18]. In addition, AI-based tools support automated organ segmentation and study prioritization, contributing to reduced reporting time and improved workflow efficiency.

In the context of value-based healthcare (VBH), radiology plays a critical role in enabling timely and accurate diagnosis, treatment planning, and monitoring of therapeutic response. AI systems may support VBH principles by improving efficiency and consistency while allowing radiologists to focus on complex decision-making and patient-centered care. Importantly, current evidence indicates that AI functions primarily as a decision-support tool rather than a replacement for radiologists, reinforcing a collaborative human-AI model.

Performance comparisons suggesting accuracy comparable to radiologists are largely derived from retrospective or controlled studies focusing on narrow diagnostic tasks and curated datasets [4,20]. Consequently, these findings should be interpreted cautiously, as generalizability across institutions, imaging devices, and patient populations remains a key challenge. Additional limitations include limited model interpretability, workflow integration barriers, and the need for prospective validation demonstrating measurable clinical outcome improvements. Linking technical advances, such as image reconstruction optimization and artifact reduction, to downstream clinical outcomes remains an important direction for future research [19].

Mammography

Screening mammography represents one of the most mature and extensively studied applications of AI in diagnostic imaging. Large retrospective studies and population-based evaluations have demonstrated that DL-based systems can increase breast cancer detection rates while reducing false-positive findings, leading to fewer unnecessary recalls and biopsies [21]. Some of the most influential evidence originates from large multicenter datasets and national screening programs, where AI has been evaluated under real-world screening conditions. In several studies, AI-assisted workflows, particularly those integrating AI as one component of double reading, have shown diagnostic performance comparable to, or in selected settings superior to, traditional human-only reading strategies [21].

However, most available evidence is derived from retrospective analyses or controlled implementations within specific geographic regions and healthcare systems, which limits generalizability. Algorithm performance may vary depending on breast density distribution, population demographics, imaging equipment, and screening protocols [4,16]. Furthermore, integration of AI into existing screening workflows raises practical challenges related to regulatory approval, radiologist acceptance, and medico-legal responsibility. Prospective studies evaluating long-term clinical outcomes, cost-effectiveness, and equitable performance across diverse populations are therefore essential to support widespread implementation.

Ophthalmology

Ophthalmology is among the first medical specialties in which AI systems have been successfully translated into large-scale clinical screening programs. DL algorithms analyzing retinal fundus photographs have demonstrated high sensitivity and specificity for detecting diabetic retinopathy, as well as other sight-threatening retinal conditions, in large retrospective datasets and prospective screening studies [22]. Meta-analyses encompassing multiethnic populations confirm that AI-based systems can achieve diagnostic performance comparable to expert ophthalmologists in well-defined screening tasks [23].

From a clinical perspective, these systems enable standardized image assessment, reduce inter-observer variability, and support scalable screening models, particularly in primary care settings and regions with limited access to ophthalmology specialists [22,23]. Nevertheless, challenges remain regarding external validation across different imaging devices, variability in image quality, and integration into established referral pathways. Ensuring appropriate clinical oversight and alignment with existing care models is critical to translating strong technical performance into improved patient outcomes.

Dermatology

In dermatology, AI has been widely investigated for the classification of skin lesions using clinical and dermoscopic images. Landmark retrospective studies using large, curated image datasets have demonstrated that CNN-based systems can achieve diagnostic performance comparable to that of experienced dermatologists in differentiating malignant from benign lesions, including melanoma [24]. These findings have driven growing interest in AI-assisted tools for triage in primary care and teledermatology settings, where early identification of high-risk lesions may substantially influence patient prognosis.

Despite promising results, important limitations have been identified. Several studies have reported reduced diagnostic accuracy in patients with darker skin phototypes, reflecting underrepresentation of diverse populations in training datasets [25]. Additional challenges include variability in image acquisition conditions and the need for prospective validation in real-world clinical environments. Addressing these limitations through diverse training data, transparent reporting, and careful clinical integration is essential to ensure equitable and clinically meaningful deployment of AI in dermatology.

Digital Pathology

Digital pathology represents a rapidly evolving domain of AI-assisted diagnostics, driven by increasing adoption of whole-slide imaging and advances in DL. AI algorithms have been applied to large histopathological image datasets for tasks such as tumor detection, grading, identification of micrometastases, and quantitative feature extraction, primarily within retrospective validation studies [5,6]. These approaches have demonstrated potential to reduce inter-observer variability among pathologists and improve diagnostic consistency, particularly in oncologic pathology.

From a clinical standpoint, AI-based tools may enhance workflow efficiency by highlighting regions of interest, supporting diagnostic prioritization, and facilitating integration of histopathological findings with molecular and clinical data [6]. However, several challenges remain, including variability in data quality, substantial computational requirements, and a lack of standardization across institutions and scanning platforms. Regulatory approval pathways and prospective studies linking AI-assisted pathology to patient management decisions and clinical outcomes remain critical for broader clinical adoption [5,6].

Comparison of AI and physician performance

Systematic reviews and meta-analyses consistently indicate that the diagnostic performance of AI algorithms in medical imaging is comparable to that of specialist physicians in clearly defined, binary classification tasks, such as disease presence versus absence [4]. A summary comparison of diagnostic performance between AI systems and specialist physicians across selected imaging-based domains is presented in Table 2. More recent evidence extending beyond early benchmark studies confirms these findings across multiple imaging-based specialties, including radiology, ophthalmology, dermatology, and digital pathology, particularly when AI systems are evaluated under controlled experimental conditions [8,23]. However, authors of these studies emphasize that the majority of available studies remain retrospective in nature, rely on curated datasets, and frequently lack external validation across diverse clinical environments [4,8].

**Table 2:** Comparison of diagnostic performance between AI systems and physicians in selected imaging-based specialties. Original table created by the authors based on published comparative studies and systematic reviews. Note: Performance comparisons are task-specific and predominantly derived from retrospective or controlled study designs; clinical impact depends on integration into real-world workflows.

Specialty	Diagnostic task	Technical performance (AI vs. physicians)	Typical study design/dataset	Relevance for patient care and clinical outcomes	Key limitations	References
Radiology	Detection of acute findings (CT, X-ray)	Comparable accuracy in narrow tasks	Retrospective validation studies on curated datasets	Faster triage of urgent cases, reduced reporting time	Limited external validation, workflow integration	[4,8,20]
Mammography	Breast cancer screening	Comparable or superior detection performance	Large retrospective and population-based studies	Improved cancer detection, reduced recall rates	Population heterogeneity, breast density variability	[21]
Ophthalmology	Diabetic retinopathy detection	Comparable or superior performance	Screening studies, large multiethnic datasets	Earlier detection, optimized referral pathways	Image quality dependency, device variability	[22,23]
Dermatology	Malignant vs benign lesion classification	Comparable performance in benchmark tasks	Retrospective image dataset studies	Improved triage in primary care and teledermatology	Skin tone bias, limited real-world validation	[24,25]
Digital pathology	Tumor detection and grading	Comparable performance	Retrospective whole-slide image analyses	Reduced inter-observer variability, workflow support	Standardization, computational demands	[5,6]

Importantly, diagnostic accuracy alone does not fully capture the clinical value of AI systems. Emerging reviews highlight that evidence regarding the impact of AI on real-world clinical decision-making, workflow efficiency, and patient-centered outcomes remains limited [2]. In routine practice, diagnostic reasoning often involves contextual factors, multimodal data integration, and longitudinal assessment, which are not fully reflected in current performance comparisons. Consequently, while AI systems may achieve parity with physicians in narrow tasks, their role should be understood as augmentative rather than substitutive, reinforcing the need for human oversight and clinical judgment.

AI as a clinical decision support system (CDSS)

In real-world clinical settings, AI most commonly functions as a CDSS, assisting clinicians by highlighting suspicious findings, prioritizing high-risk cases, and providing supplementary diagnostic or prognostic information [2,6]. Evidence from observational studies and prospective evaluations suggests that AI-based CDSS can improve workflow efficiency by reducing reporting time, supporting triage of urgent cases, and decreasing clinician workload in high-volume environments such as radiology and laboratory medicine [9-11]. In selected applications, CDSS have also been associated with reduced diagnostic error rates and improved adherence to clinical guidelines, particularly when used as adjuncts rather than autonomous systems.

Despite these potential benefits, real-world deployment of CDSS is associated with important limitations. Automation bias - the tendency of clinicians to over-rely on algorithmic recommendations - remains a well-documented risk and may negatively affect decision quality if AI outputs are accepted uncritically [26]. Additional challenges include variability in system performance across institutions, limited generalizability to new patient populations, and difficulties integrating CDSS into existing clinical workflows and electronic health record systems.

Effective implementation, therefore, requires a human-centered integration strategy. This includes maintaining clinicians as final decision-makers, providing transparent and interpretable system outputs, and ensuring adequate training to promote appropriate interaction with AI tools. Gradual integration into diverse clinical environments, combined with user education and continuous performance monitoring, is essential to maximize clinical benefit while minimizing unintended consequences associated with CDSS deployment [1,17].

Limitations, ethical, and regulatory aspects

Despite substantial progress, several ethical, technical, and regulatory challenges continue to limit the safe and effective implementation of AI in clinical practice. A central technical concern is algorithm generalizability, as AI systems trained on data from specific institutions, devices, or patient populations may demonstrate degraded performance when deployed in new clinical environments [4,7,16]. For example, diagnostic algorithms validated on curated imaging datasets have been shown to underperform when applied to real-world clinical data characterized by greater variability in image quality and patient demographics [4,16].

Ethical challenges extend beyond technical performance and frequently arise at the point of clinical deployment. Algorithmic bias remains a significant concern, particularly when underrepresented patient groups are insufficiently represented in training datasets, potentially exacerbating existing healthcare disparities [16]. In addition, limited model interpretability in so-called “black-box” systems complicates clinical accountability and may reduce clinician trust, especially in high-stakes decision-making contexts [17]. Practical mitigation strategies include the use of diverse and representative training data, external validation across multiple sites, and the incorporation of explainable AI approaches to support clinician understanding and oversight [16,17].

Regulatory frameworks governing medical AI are evolving in response to these challenges, emphasizing risk-based classification, human oversight, and post-market performance monitoring [27,28]. However, tensions may arise between ethical principles, regulatory requirements, and clinical utility. For instance, strict regulatory constraints may limit the adaptive updating of learning algorithms, potentially reducing clinical relevance over time, while insufficient regulation may expose patients to unvalidated or biased systems [17,27]. Balancing innovation with patient safety, therefore, requires close collaboration between clinicians, developers, and regulators, as well as continuous evaluation of AI systems in real-world clinical settings [16,17,28]. Collectively, these considerations underscore that ethical and regulatory governance must be integrated with, rather than treated as separate from, assessments of clinical effectiveness when implementing AI in healthcare (Table 3).

**Table 3:** Key limitations, ethical challenges, and regulatory considerations in the clinical implementation of artificial intelligence (AI). Original table created by the authors based on published literature, consensus recommendations, and regulatory guidance documents. EU AI Act: European Union Artificial Intelligence Act; FDA: Food and Drug Administration; SaMD: Software as a Medical Device

Domain	Key issues	Clinical implications	Mitigation strategies	References
Technical limitations	Limited generalizability, dataset shift	Reduced performance in new clinical settings	External validation, multicenter datasets	[4,7,16]
Algorithmic bias	Underrepresentation of patient subgroups	Risk of health disparities, inequitable care	Diverse training data, bias audits	[16]
Human-AI interaction	Automation bias, overreliance on AI	Diagnostic or decision-making errors	Human-in-the-loop design, clinician training	[17,26]
Transparency	Black-box models, limited explainability	Reduced trust, accountability challenges	Explainable AI methods, model documentation	[16,17]
Data privacy	Large-scale data use, re-identification risk	Breaches of confidentiality	Privacy-preserving learning, governance frameworks	[16,17]
Regulation	Evolving legal frameworks, liability issues	Unclear accountability, delayed adoption	EU AI Act, FDA SaMD guidance	[27,28]

The role and applications of AI in clinical laboratory diagnostics

AI is increasingly being integrated into clinical laboratory diagnostics, where growing test volumes and data complexity challenge traditional analytical approaches. AI-based systems have been applied to multiple stages of laboratory workflows, including sample quality assessment, microscopic image analysis, result interpretation, and clinical decision support [9,10]. Quantitative evidence indicates that AI-assisted laboratory tools can improve diagnostic accuracy and workflow efficiency. For example, ML models applied in hematology and biochemistry have demonstrated high sensitivity and specificity in identifying abnormal samples and clinically significant result patterns, while prospective cohort studies have reported reductions in turnaround time and improved prioritization of urgent cases [9-11].

In addition, AI-driven CDSS in laboratory medicine have been shown to enhance result interpretation and reduce unnecessary follow-up testing. In a prospective evaluation of an AI-based LabTest Checker system, high diagnostic accuracy was observed in both routine and urgent case classification, suggesting potential benefits for patient safety and clinician workload reduction [11]. Collectively, these applications highlight the capacity of AI to support laboratory professionals by automating repetitive tasks, improving consistency, and facilitating timely clinical decision-making.

Despite these advantages, important limitations remain. Algorithm performance may be affected by variability in laboratory equipment, analytical methods, and patient populations, raising concerns regarding generalizability across diverse laboratory settings [9,10]. Furthermore, many AI systems have been validated in single-center or controlled environments, underscoring the need for external, multi-center validation. Integration with existing laboratory information systems and clinical workflows also presents practical challenges that may influence adoption and real-world effectiveness. Addressing these limitations through standardized validation, interoperability-focused system design, and prospective outcome-based studies will be essential to support broader clinical implementation of AI in laboratory diagnostics.

Integrating AI into rehabilitation practices

AI has been increasingly explored as a supportive tool in rehabilitation, driven by growing demand for personalized and resource-efficient therapeutic interventions in aging populations and individuals with chronic conditions [12-14]. While early studies suggest that AI-enabled rehabilitation technologies - including robotic-assisted therapy, motion analysis systems, and adaptive digital platforms - may support therapy personalization, objective feedback, and patient engagement, the current evidence base remains heterogeneous and characterized by variable effect sizes [12-14]. Most reported benefits are modest and context-dependent, often derived from small or short-term studies, underscoring that AI is best viewed as an adjunct to conventional rehabilitation rather than a standalone therapeutic solution [12,14].

Stroke

AI has emerged as a promising adjunct in post-stroke rehabilitation, particularly for addressing persistent upper limb impairments that limit functional independence [12,29]. Evidence from systematic reviews and randomized controlled trials suggests that AI-assisted and robot-supported interventions may lead to improvements in motor function, including enhanced Fugl-Meyer scores and active range of motion, compared with conventional therapy alone [12,29]. However, most available studies are characterized by relatively small sample sizes, short intervention periods, and limited follow-up, which constrain conclusions regarding long-term functional recovery and quality-of-life outcomes [12,29].

Practical challenges also influence real-world implementation, including the cost of robotic systems, infrastructure requirements, and the need for clinician training [12]. Accessibility remains limited in low-resource settings, and algorithm performance may vary across patient populations with differing stroke severity or comorbidities, raising concerns regarding generalizability [12,29]. Consequently, while AI-driven stroke rehabilitation shows potential to enhance functional outcomes, further large-scale prospective studies are required to confirm sustained benefits, cost-effectiveness, and applicability across diverse clinical contexts [12,29].

Low Back and Neck Pain

AI-based digital interventions have been explored as adjuncts to conventional rehabilitation for low back and neck pain, primarily through personalized self-management platforms [30,31]. Strategies to enhance patient engagement include individualized exercise recommendations, adaptive educational content, and continuous feedback, which have been shown to influence adherence and perceived usefulness of digital interventions [30]. Clinical effectiveness appears to be modulated by factors such as baseline symptom severity, patient motivation, and integration with standard rehabilitation pathways rather than use as a standalone intervention [30,31].

While randomized trials have not consistently demonstrated superior short-term clinical outcomes compared with usual care, AI-driven personalization offers opportunities to optimize exercise selection, progression, and adherence [31]. Future refinements focusing on closer integration with supervised rehabilitation, improved engagement strategies, and alignment with measurable functional outcomes may enhance the clinical impact of these interventions [30,31].

Knee Osteoarthritis

Recent studies suggest that large language models may support the preliminary design of individualized rehabilitation programs for knee osteoarthritis by generating exercise plans aligned with structured clinical assessments [32]. In routine practice, such tools would require ongoing oversight by physiotherapists to ensure patient safety, appropriate exercise progression, and adherence to evidence-based rehabilitation principles [32]. AI-generated programs may assist clinicians by streamlining planning processes; however, they should not replace individualized clinical reasoning or professional judgment [32].

Current evidence is limited by observational study designs, modest sample sizes, and the absence of prospective validation, which restricts conclusions regarding long-term effectiveness and generalizability [32]. Variability in disease severity, functional capacity, and comorbidities further underscores the need for cautious implementation and future studies assessing patient outcomes, adherence, and quality of life within supervised clinical workflows [32].

Cardiac Rehabilitation

AI has been applied to technology-assisted cardiac rehabilitation to analyze patient feedback, adherence, and functional performance using natural language processing approaches [33]. In the referenced observational study, AI-derived insights from patient-reported experiences were correlated with objective measures of exercise capacity, suggesting a potential relationship between patient engagement and functional improvement [33]. However, the study design and limited sample size constrain statistical robustness and preclude causal inference regarding clinical effectiveness [33].

From an implementation perspective, AI-driven monitoring tools may support routine cardiac rehabilitation by enhancing engagement and identifying individuals at risk of non-adherence [33]. Nevertheless, broader adoption requires validation across diverse patient populations and care settings, integration with existing rehabilitation workflows, and evaluation of long-term clinical outcomes [33]. Considerations related to digital literacy, clinician oversight, and scalability remain critical for successful real-world implementation [33].

Sports Rehabilitation

AI-supported systems have been explored in long-term sports rehabilitation and health management through integration of wearable devices, cloud-based platforms, and personalized feedback mechanisms [34]. While reported improvements in physiological parameters, health knowledge, and adherence are encouraging, available evidence is limited by moderate sample sizes, variable study duration, and restricted population diversity [34]. These limitations reduce the ability to generalize findings to broader athletic or clinical populations and to assess sustained functional benefits [34].

Further prospective studies with larger and more diverse cohorts are required to evaluate long-term outcomes, adherence, and comparative effectiveness relative to conventional rehabilitation approaches [34].

Spinal Cord Injury

AI-based motion analysis systems have been developed to support upper extremity rehabilitation in patients with spinal cord injury by providing real-time visual feedback during exercise [35]. Although preliminary randomized trials suggest improvements in muscle strength and exercise execution, statistical power is limited, and between-group differences often do not reach significance [35]. Strategies to enhance effect sizes may include longer intervention periods, adaptive progression algorithms, and combination with supervised rehabilitation [35].

Scalability and accessibility remain key considerations, particularly for home-based rehabilitation programs [35]. Successful implementation requires patient training, reliable access to technology, and sustained adherence, all of which may influence clinical effectiveness [35]. Further research is needed to assess long-term functional outcomes, feasibility in home settings, and cost-effectiveness of AI-assisted rehabilitation for individuals with spinal cord injury [35].

Chatbots

Rising demand for healthcare services, together with advances in AI, has driven the development of conversational agents intended to support a wide range of health-related tasks [15]. The use of chatbots has the potential to improve public access to quality health care [36].

Complementary Roles of Chatbots and Physicians

The question arises whether an AI chatbot can provide responses to patient inquiries that match the quality and empathy of those written by physicians. In a study analyzing 195 patient questions randomly selected from a social media forum, licensed healthcare professionals compared responses provided by physicians with those generated by a chatbot. Responses generated by the chatbot were favored over those of physicians and received significantly higher scores for both quality and empathy. These outcomes suggest that AI-based systems have the potential to help draft responses for patient inquiries [37]. The rapid growth of virtual healthcare has led to an increase in patient messages, contributing to higher workloads and burnout among healthcare professionals. AI assistants may support the creation of answers to patient questions by drafting replies for subsequent review by clinicians, potentially alleviating workload while maintaining response quality [36]. This cross-sectional study shows promising results for AI assistants in responding to patient questions, but further research is needed to confirm their clinical impact. Despite study limitations and the tendency to overhype new technologies, integrating AI assistants into patient messaging workflows may enhance outcomes for both clinicians and patients [36,37].

Mental Health

Recent years have shown a rise in stress and mental health disorders within the general population, increasing demand for accessible and scalable mental health interventions [36]. Digital interventions using chatbots have emerged as a promising tool to support mental well-being, offering continuous availability, personalization, and low-threshold access to support [36]. Chatbot-based interventions may support improvements in mindfulness and emotion regulation among stressed individuals, as demonstrated in randomized controlled trials evaluating digital mental health interventions [38]. Further investigation is warranted into factors such as participants’ social motivation toward chatbot guidance and the chatbot’s personality, as these may enhance the therapeutic alliance between the user and the system. Future research should explore which components of the intervention, including psychoeducation and structured exercises, contribute most to improving different mental health outcomes. In this context, large language models represent a promising avenue for the future development and refinement of AI-based chatbots for digital mental health interventions [38].

Depression and Anxiety

Depression is a significant concern among young adults, and chatbots have emerged as a widely used intervention tool. While social cues are incorporated into chatbot design, their impact on depression treatment remains under investigation. A study was conducted to compare the effectiveness of a high-social-cue (HSC) therapeutic chatbot, which included voice, facial animations, and gestures, with a low-social-cue (LSC) text-only version in providing self-help depression support for college students. Findings indicated that the HSC chatbot led to greater reductions in depressive symptoms and a stronger therapeutic connection compared to the LSC version [39].

Another study focused on developing a locally tailored AI chatbot and evaluating its effectiveness in reducing anxiety and depression among individuals in Hong Kong, compared to a conventional nurse hotline. The findings indicated that the AI chatbot performed similarly to the nurse hotline in alleviating participants’ anxiety and depression following their inquiries. Additionally, the chatbot demonstrated potential for reducing short-term anxiety and depressive symptoms, suggesting that AI-based interventions could serve as a viable supplement to traditional mental health support services. These results indicated that AI chatbots could function as a supplementary resource, providing accessible mental health support to populations with limited access to conventional care. The study was constrained by a small sample, which may affect the generalizability of the findings. Furthermore, the duration of the study was limited, which restricted participant recruitment. However, preliminary findings indicated a notable decrease in anxiety and depression following the use of the AI chatbot, highlighting its potential as a tool for mitigating negative emotions especialy during future epidemic crises [40].

Eating Disorders

Early intervention is essential for improving the prognosis of eating disorders. Single-session interventions (SSIs) may offer short-term support for individuals awaiting formal treatment. However, access to SSIs is not always readily available. Eating disorders are complex conditions characterized by abnormal eating patterns that affect both physical and psychosocial functioning. They represent a significant global health and economic burden, and addressing associated medical and psychiatric risks is a critical component of treatment. Early intervention is considered best practice, as delays can exacerbate symptoms and increase the risk of treatment dropout. Consequently, temporary support measures are needed for individuals awaiting treatment. A chatbot-delivered single-session intervention for older adolescents and adults awaiting outpatient treatment effectively reduced eating disorder symptoms, psychosocial impairment, depression, and anxiety, while demonstrating high usability and increasing the likelihood of engaging in in-person treatment. The eating disorder electronic SSI may offer an effective, accessible, convenient, and scalable early intervention for individuals awaiting eating disorder treatment. Nevertheless, additional research is needed involving more diverse populations and extended follow-up periods [41].

Health Education Among Oncology Patients

AI chatbots may be used in health education due to their round-the-clock availability, personalization, and interactive features. The trial evaluated the impact of an AI chatbot intervention on knowledge, empowerment, and attitudes toward AI among breast cancer patients. Women were randomly assigned to receive either AI chatbot-based education plus standard care or standard care alone. Outcomes were assessed using validated questionnaires measuring breast cancer and AI knowledge, attitudes toward AI, and perceived empowerment. After the intervention, participants in the intervention group demonstrated greater knowledge and more positive attitudes than those in the control group. AI chatbots in oncology nursing enhance patient knowledge, empowerment, and acceptance, highlighting their role in patient-centered digital health strategies [42].

Discussion

The present narrative review highlights the expanding and increasingly nuanced role of AI across multiple domains of healthcare, including diagnostic imaging, laboratory medicine, rehabilitation, and patient-centered digital health solutions [1,2]. Collectively, the reviewed evidence demonstrates that AI-based systems can achieve high technical performance in narrowly defined clinical tasks, particularly those reliant on pattern recognition and large-scale data analysis [4,8]. These capabilities position AI as a valuable adjunct in healthcare systems facing increasing diagnostic demand, workforce shortages, and growing complexity of clinical decision-making [1,2,7]. However, performance metrics alone do not fully capture the clinical value of AI, and their interpretation must be contextualized within real-world implementation, safety, and ethical considerations [2,16].

In imaging-based specialties, the clinical promise of AI is most clearly demonstrated in applications such as mammography screening, ophthalmic disease detection, and selected radiological tasks. In mammography, large population-based studies indicate that AI-assisted double-reading strategies may improve cancer detection rates while reducing false-positive findings and radiologist workload [21]. In ophthalmology, AI-enabled screening for diabetic retinopathy has shown robust diagnostic accuracy and has already been implemented in large-scale screening programs, enabling earlier detection and more efficient referral pathways [22,23]. In radiology and digital pathology, AI systems support lesion detection, image segmentation, and workflow optimization, contributing to efficiency gains and improved diagnostic consistency [5-7,18]. Across these domains, AI is best understood as a decision-support technology that complements, rather than replaces, clinician expertise [1,17].

Beyond diagnostic imaging, AI applications in laboratory medicine, rehabilitation, and digital health illustrate a broader clinical utility. In laboratory diagnostics, AI-based systems support result interpretation, workflow optimization, and clinical decision support, addressing the growing volume and complexity of diagnostic data [9-11]. In rehabilitation, AI-enabled tools - including robotic systems, motion analysis platforms, and large language models - facilitate personalized therapy planning, objective progress monitoring, and patient engagement, with evidence derived from systematic reviews, randomized controlled trials, and observational studies [12-14,29]. Similarly, conversational agents and digital health applications show promise in patient education, mental health support, and chronic disease management, although the quality and consistency of available evidence remain variable [15,36-42].

Despite substantial progress, several overarching challenges continue to limit the widespread clinical adoption of AI. Algorithm generalizability remains a central concern, as models trained on specific populations or technical environments may underperform when deployed in new clinical settings [4,16]. Algorithmic bias, automation bias, and limited interpretability of “black-box” systems highlight the necessity of maintaining clinician oversight and accountability [16,17,26]. Ethical considerations - including transparency, data privacy, and patient safety - are inseparable from technical performance and directly influence trust in AI-enabled healthcare [16,17]. Regulatory frameworks, such as the European Union Artificial Intelligence Act and guidance on AI/ML-based Software as a Medical Device, are evolving to address these challenges; however, tensions between innovation, adaptability, and patient safety persist [27,28].

Taken together, the strength of available evidence supporting AI applications varies considerably across clinical domains and should be interpreted in relation to study design and clinical context. In imaging-based specialties such as radiology, mammography, ophthalmology, dermatology, and digital pathology, most performance claims are derived from retrospective analyses or controlled validation studies, frequently based on curated datasets (Tables 1-2) [4,8,21-25]. While these studies consistently demonstrate high technical accuracy for narrowly defined diagnostic tasks, prospective evaluations assessing real-world clinical impact remain limited [4,8]. In contrast, selected applications in rehabilitation and digital health - including AI-assisted therapy and chatbot-based interventions - are supported by randomized controlled trials; however, these studies are often small, heterogeneous, and focused on short-term outcomes (Table 1) [12-14,29,38-41].

Importantly, technical performance alone does not equate to clinical benefit. As summarized in Table 2, AI systems demonstrating accuracy comparable to healthcare professionals appear to offer the greatest clinical value when deployed as decision-support tools that enhance workflow efficiency, support triage, and reduce cognitive burden, rather than as autonomous systems [2,4]. Nevertheless, evidence directly linking AI implementation to improved patient-centered outcomes, long-term health benefits, or cost-effectiveness remains sparse across most domains [2]. Table 3 further highlights that limitations related to generalizability, algorithmic bias, human-AI interaction, and evolving regulatory requirements continue to pose substantial barriers to widespread clinical adoption [16,17,26-28].

From an implementation perspective, successful integration of AI into routine clinical practice requires alignment with existing clinical workflows, interoperability with electronic health record systems, and adequate clinician training [1,17]. Evidence suggests that insufficient user training, poor workflow integration, and lack of institutional readiness may limit adoption or even negate potential benefits of AI systems [2,26]. Cost-effectiveness considerations - including implementation costs, system maintenance, and regulatory compliance - are also critical yet remain insufficiently addressed in current evaluations [2]. Addressing these gaps through prospective, multi-center studies that incorporate clinical outcomes, economic analyses, and implementation science frameworks represents a key priority for advancing the responsible and effective use of AI in healthcare.

## Conclusions

AI represents a transformative and rapidly evolving component of modern healthcare, with meaningful applications across medical diagnostics, laboratory medicine, rehabilitation, and patient-centered digital health solutions. Evidence across multiple domains indicates that AI-based systems can enhance diagnostic accuracy in selected, well-defined tasks, improve workflow efficiency, and support more personalized approaches to treatment and rehabilitation. However, current findings are largely derived from retrospective analyses, controlled validation studies, or small-scale trials, underscoring that AI should be regarded as a complementary technology rather than a replacement for clinical expertise.

The safe and effective integration of AI into routine clinical practice will depend on prospective, real-world validation studies that assess not only technical performance but also patient-centered outcomes, safety, cost-effectiveness, and implementation feasibility. In addition, interdisciplinary collaboration among clinicians, data scientists, engineers, ethicists, and regulatory stakeholders will be essential to ensure that AI systems are developed and deployed responsibly, transparently, and in alignment with clinical workflows and patient needs. When supported by rigorous validation and collaborative implementation, AI holds substantial potential to improve the quality, efficiency, and accessibility of healthcare.
